# Mosquitoborne Sindbis Virus Infection and Long-Term Illness

**DOI:** 10.3201/eid2406.170892

**Published:** 2018-06

**Authors:** Åsa Gylfe, Åsa Ribers, Oscar Forsman, Göran Bucht, Gerd-Marie Alenius, Solveig Wållberg-Jonsson, Clas Ahlm, Magnus Evander

**Affiliations:** Umeå University, Umeå, Sweden (Å. Gylfe, Å. Ribers, O. Forsman, G.-M. Alenius, S. Wållberg-Jonsson, C. Ahlm, M. Evander);; Swedish Defense Research Agency, Umeå (G. Bucht)

**Keywords:** Sindbis virus, Ockelbo disease, chronic arthritis, outbreak, Sweden, alphavirus, vector-borne infections, viruses, *Suggested citation for this article*: Gylfe Å, Ribers Å, Forsman O, Bucht G, Alenius G-M, Wållberg-Jonsson S, et al. Mosquitoborne Sindbis virus infections and long-term illness. Emerg Infect Dis. 2018 Jun [*date cited*]. https://doi.org/10.3201/eid2406.170892

## Abstract

An unexpected human outbreak of the mosquitoborne Sindbis virus occurred in a previously nonendemic area of Sweden. At follow-up, 6–8 months after infection, 39% of patients had chronic arthralgia that affected their daily activities. Vectorborne infections may disseminate rapidly into new areas and cause acute and chronic disease.

Mosquitoborne viruses such as chikungunya virus, Ross River virus, and Sindbis virus (SINV) are members of the genus *Alphavirus* (family *Togaviridae*) and cause human arthritic diseases (*1*). SINV has mainly been reported in northern Europe and South Africa (*1*); Sweden has an average of 3 SINV cases per year, with occasionally more cases in a previously defined endemic region in central Sweden (Figure) (*2*). Birds are the reservoir for SINV, and there is no evidence of human-to-human transmission. SINV infection in humans, called Ockelbo disease in Sweden, causes rash, arthritis, and mild fever (*3**–**5*). Most patients recover within weeks or months, but arthralgia and myalgia can persist for years following infection, suggesting inflammatory response or a persistent infection (*4**–**6*).

**Figure:**
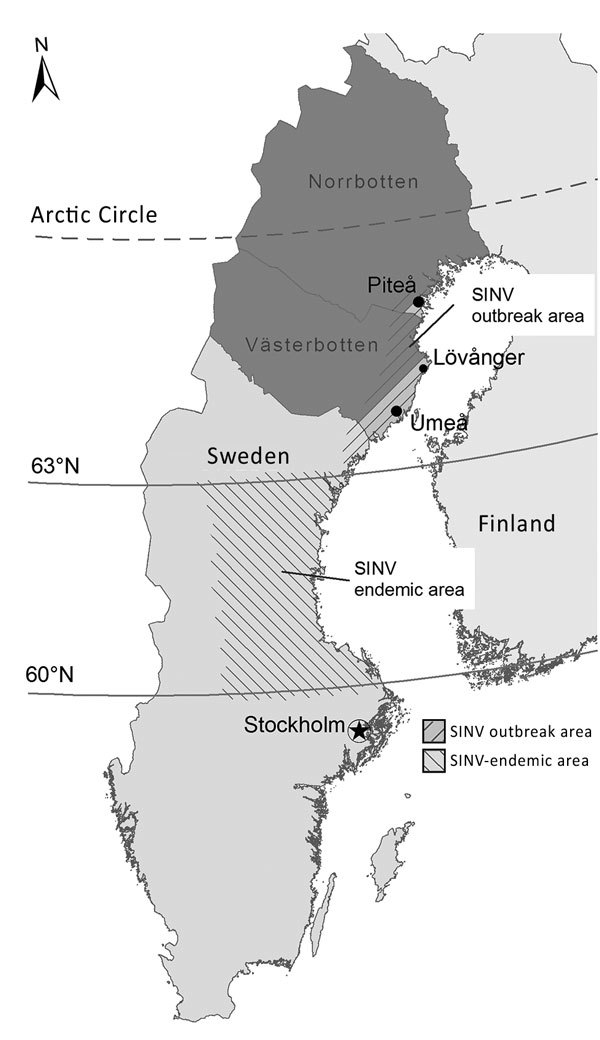
Geographic distribution of the SINV outbreak in 2013 and previous occurrence of SINV infections in Sweden. Dark gray indicates the 2 northernmost counties in Sweden where the SINV IgG seroprevalence was 2.9% in 2009. SINV, Sindbis virus.

In mid-August 2013, several patients with rash, arthralgia, and fever visited the healthcare center in the small village of Lövånger in Västerbotten County, Sweden (Figure). The university hospital laboratory in Umeå received 172 blood samples from patients with suspected SINV infection; 50 patients had SINV-specific IgM and IgG (Technical Appendix). SINV infections have been believed to be almost exclusively confined to the central part of Sweden (*2*), but the 2013 outbreak occurred north of the endemic area (Figure). The distribution of verified cases by sex in this outbreak showed a higher proportion of female patients (62%) than male patients (38%) with acute SINV infection (Technical Appendix Table 1).

Previous reports suggested that joint symptoms might persist for years in SINV infections (*4**–**6*). To evaluate long-term consequences, we contacted 46 SINV patients by telephone 3–4 months, 6–8 months, or in both periods after acute disease (Technical Appendix Figure 1). Our study was approved by the Regional Ethics Review Board (2014-102-32M), and we obtained written informed consent from all participants. We include details of the study results summarized here in the Technical Appendix. In total, 18/46 (39%) of the patients reported persistent musculoskeletal pain (arthralgia and myalgia) and restriction in their daily activity 6–8 months after the onset of acute symptoms (Technical Appendix Table 2). We invited these patients for a standardized examination by a rheumatologist, including a health assessment questionnaire, patient assessments of their pain and their global health using a visual analog scale, and a blood sample (Technical Appendix). Of 17 symptomatic patients who participated, 1 had arthritis in the ankle, 14 had >1 tender joint, and 10 had enthesitis, tendinitis, or tenosynovitis at examination. Large joints (knee, hip, shoulder, wrists, ankles) and small joints (toes, fingers) were affected, with a predominance for the lower extremities, in contrast to other studies in which small and peripheral joints were mainly affected (*4**–**6*). Patients graded their global symptoms as more severe than the examining doctor did, indicating that joint function was only mildly affected whereas the pain was perceived as restricting. Test results did not detect citrulline antibodies, and the single patient with positive rheuma factor had no arthritic symptoms. A notable finding was that 4 patients (24%) had psoriasis, a condition present in <4% of the northern European population (*7*), raising the question whether psoriasis makes SINV patients more vulnerable to long-term arthralgia. 

We asked the 28 patients at the 6- to 8-month follow-up who had recovered to complete a questionnaire and donate a blood sample at their local healthcare provider; 23 did so. Symptomatic patients reported more pain and impaired health, compared with patients who were asymptomatic 6–8 months after acute disease. We detected SINV-specific IgM in patients with and without persistent symptoms, as previously reported (*5*).

We isolated a new SINV strain from a mosquito caught in the area during the outbreak; it was most closely related to a SINV strain from Finland (*8*). However, no increased incidence was recorded in Finland either the year before or concomitantly with the Swedish outbreak (*9*). In addition, only 1 case was reported from the endemic area of central Sweden in 2013, suggesting that local factors such as weather conditions may determine an outbreak. June 2013 stood out with a high mean temperature and precipitation, which have been shown to be associated with a high incidence of SINV infection later in summer (Technical Appendix Figure 2). 

A recent study in northern Sweden revealed that in a randomly selected population-based cohort, 2.9% had SINV IgG (Figure), indicating that the virus was present in the region, although not recognized (*10*). More research is warranted regarding the long-lasting joint pain caused by a previous SINV infection; patients with undiagnosed SINV may visit a healthcare facility with such symptoms even several months postinfection. Our report illustrates how a vectorborne zoonotic disease can result in a large, unexpected outbreak. The key factors for outbreaks of SINV or other alphavirus-caused diseases are generally unknown, which warrants further investigations, especially in light of the global emergence of alphaviruses (*1*).

Technical Appendix Additional information and study findings of Sindbis virus infection, Sweden, 2013.
