# Supplementary material for: Mosquitoborne Sindbis Virus Infection and Long-Term Illness
Source: Emerg Infect Dis. 2018 Jun;24(6):1141–2. doi: 10.3201/eid2406.170892 (PMC6004841; doi:10.3201/eid2406.170892)
Supplement: Technical Appendix — Additional information and study findings of Sindbis virus infection, Sweden, 2013. [file 17-0892-Techapp-s1.pdf]

Article DOI: https://doi.org/10.3201/eid2406.170892

Mosquitoborne Sindbis Virus Infection and Long-Term Illness

Technical Appendix

Case Definition

We selected cases on the basis of the patient samples that were sent to the clinical microbiology laboratory at Norrland University Hospital, Umeå, Sweden for Sindbis virus (SINV) diagnosis during August–October 2013. We analyzed all samples for IgG and IgM SINV antibodies. Case-patients either had IgM and IgG antibodies against SINV in a single serum sample or showed seroconversion in a follow-up sample. The clinicians at local healthcare centers in northern Sweden referred serum samples to the laboratory based on the combined acute symptoms of joint pain, rash, and sometimes fever, muscle pain, or both. The healthcare centers had been alerted by the Västerbotten County Center for Disease Control, who had sent out a request to look for patients with these acute symptoms, because there had been many cases in a short time and it was not known at that time which agent caused the symptoms.

Immunofluorescence Assay

We analyzed the presence of SINV-specific antibodies in the blood samples with an indirect immunofluorescence assay (IFA) (*1*) used in routine diagnostics at Norrlands University hospital, Umeå Sweden. The antigen was SINV Edsbyn 5/82 (*2*) cultured to 60%–70% cytopathogenic effect in Green Monkey Kidney cells in Dulbecco´s Modified Eagle´s Medium (Sigma-Aldritch, St. Louis, MO, USA) supplemented with 2% Fetal Bovine Serum (Sigma-Aldritch). We harvested infected cells with a cell scraper, washed them twice in phosphate buffered saline (PBS) pH 7.4 and spotted to 70% confluence in 6 mm wells on Teflon-coated glass slides. We allowed slides to dry at room temperature overnight, fixed them in acetone, and stored them at −70°C for later use in IFA. We diluted serum samples 1:10 and 1:40 in PBS and incubated them on the glass slides for 1 hour at 37°C. After washing for 10 minutes in PBS, we

incubated the slides for 1 hour at 37°C with polyclonal rabbit anti-human FITC conjugated IgG or IgM (DAKO, Denmark). After rinsing slides with PBS for 10 minutes, we mounted them with glycerol for fluorescence microscope examination to determine specific granular fluorescence. We included a positive control (human serum previously tested positive for SINV-antibodies) in every batch. We verified all serum samples that were reactive in the IFA with a SINV-specific EIA. To compare the performance of the IFA, we analyzed samples by both methods, 172 samples for IgG and 167 samples for IgM analysis. For IgG detection, the IFA had 76.2% sensitivity and 99.1% specificity compared with the EIA. For IgM detection, the IFA had 96.1% sensitivity and 81.7% specificity compared with the EIA.

ELISA

We measured specific IgG against SINV by an enzyme immunoassay (EIA) as previously described (*3*). Briefly, the SINV antigen, we water-sonicated SINV Lövånger 2013 (*4*) on ice 4 times for 30 s each and box-titrated it to determine the optimal antigen dilution. We incubated diluted serum samples (1:420) at 4°C overnight on antigen-coated wells, washed 4 times with PBS-T, again incubated for 60 min at 37°C with 100 µL goat anti-human IgG alkaline phosphate conjugate (Invitrogen Corporation, USA, lot: 722339A) (1:6000). We added substrate (p-nitrophenyl phosphate disodium; Sigma Diagnostics, USA) and incubated for 30 min at 37°C; we recorded the optical density (OD) at 405 nm. We subtracted the mean OD of 8 blank wells (containing PBS-T and 1% milk) from each result. We determined the cutoff in SINV IgG EIA by analyzing 32 serum samples previously confirmed SINV negative by immunofluorescence assay. We preliminarily determined the assay cutoff by counting the mean OD value of these SINV negative samples +3 standard deviations. We analyzed all serum samples once and those with an OD above cutoff were analyzed again in duplicate. The SINV antibody-positive results from the EIA had previously been verified using a SINV-specific haemagglutination inhibition (HI) test (*3*,*5*).

References

1. Espmark A, Niklasson B. Ockelbo disease in Sweden: epidemiological, clinical, and virological data from the 1982 outbreak. Am J Trop Med Hyg. 1984;33:1203–11. PubMed http://dx.doi.org/10.4269/ajtmh.1984.33.1203

2. Niklasson B, Espmark A, LeDuc JW, Gargan TP, Ennis WA, Tesh RB, et al. Association of a Sindbis-like virus with Ockelbo disease in Sweden. Am J Trop Med Hyg. 1984;33:1212–7. PubMed http://dx.doi.org/10.4269/ajtmh.1984.33.1212

3. Ahlm C, Eliasson M, Vapalahti O, Evander M. Seroprevalence of Sindbis virus and associated risk factors in northern Sweden. Epidemiol Infect. 2014;142:1559–65. PubMed http://dx.doi.org/10.1017/S0950268813002239

4. Bergqvist J, Forsman O, Larsson P, Näslund J, Lilja T, Engdahl C, et al. Detection and isolation of Sindbis virus from mosquitoes captured during an outbreak in Sweden, 2013. Vector Borne Zoonotic Dis. 2015;15:133–40. PubMed http://dx.doi.org/10.1089/vbz.2014.1717

5. Manni T, Kurkela S, Vaheri A, Vapalahti O. Diagnostics of Pogosta disease: antigenic properties and evaluation of Sindbis virus IgM and IgG enzyme immunoassays. Vector Borne Zoonotic Dis. 2008;8:303–12. PubMed http://dx.doi.org/10.1089/vbz.2007.0623

6. Swedish Meteorological and Hydrological Institute. Meteorological observations [in Swedish]. http://opendata-download-metobs.smhi.se/explore/

7. Jalava K, Sane J, Ollgren J, Ruuhela R, Rätti O, Kurkela S, et al. Climatic, ecological, and socioeconomic factors as predictors of Sindbis virus infections in Finland. Epidemiol Infect. 2013;141:1857–66. PubMed http://dx.doi.org/10.1017/S095026881200249X

8. Ekdahl C, Eberhardt K, Andersson SI, Svensson B. Assessing disability in patients with rheumatoid arthritis: use of a Swedish version of the Stanford Health Assessment Questionnaire. Scand J Rheumatol. 1988;17:263–71. PubMed http://dx.doi.org/10.3109/03009748809098795

Technical Appendix Table 1. Laboratory diagnosis Sindbis virus in patients, Sweden, August–October 2013*

Characteristics	No. IgM and IgG positive	Median age, y (range)
Patient's sex (%)		
F	31 (62)	53 (33–85)
M	19 (38)	55 (28–72)
Geographic area		
Nonendemic area†	49	NA
Endemic area‡	1	NA

*50 patients had SINV-specific IgM and IgG in a single or a follow-up sample. NA, not applicable.
†The area in Sweden north of the 63rd parallel.
‡The area in Sweden between the 60th and 63rd parallels.

Technical Appendix Table 2. Follow-up information for patients with Sindbis virus 6–8 months after the outbreak, Sweden, 2014*

Characteristic	No. patients with persistent arthralgia/myalgia (n = 17)	No. patients who recovered (n = 23)
Patient characteristics		
Sex, no. (%)		
F	9 (53)	12 (52)
M	8 (47)	11 (48)
Median age, y (range)	54 (40–72)	ND
Clinical findings, no. (%)		
Arthritis in any joint of 66 joints assessed	1 (5.9)	ND
Any tender joint of 68 joints assessed	14 (82.4)	ND
Enthesitis/tendinitis/tenosynovitis	10 (58.8)	ND
Median no. tender joints/patient (range)	3 (0–38)	ND
Laboratory findings		
SINV IgM, no. (%)†	11 (65)	14 (61)
Rheuma factor positive, no. (%)‡	1 (5.9)	ND
Anti-citrullinated protein antibodies, no. (%)	0 (0)	ND
C-reactive protein (mg/L), median (range*)*	1 (0.6–3.4)	ND
Erythrocyte sedimentation rate (mm/h), median (range)	5 (1–21)	ND
Self-graded symptoms		
Pain VAS, mm§	36	10
Global disease activity VAS, mm§	31	6
Fatigue VAS, mm§	38	26
Health assessment questionnaire, 0–3 scale¶	0.38	0
Doctor´s global VAS, mm#	11	ND

*mm, millimeters; ND, not determined; VAS, visual analog scale
†IgM levels analyzed by enzyme immunoassay.
‡Rheuma factor >3.5 IU/mL is positive.
§Visual analog scale range 0–100 mm: pain VAS (patient assessment of pain), global disease activity VAS (patient's assessment of global health), fatigue VAS (patient's assessment of fatigue).
¶Health assessment questionnaire to investigate the patient's assessment of function (*8*).
#Doctor's assessment of the patient's global health.

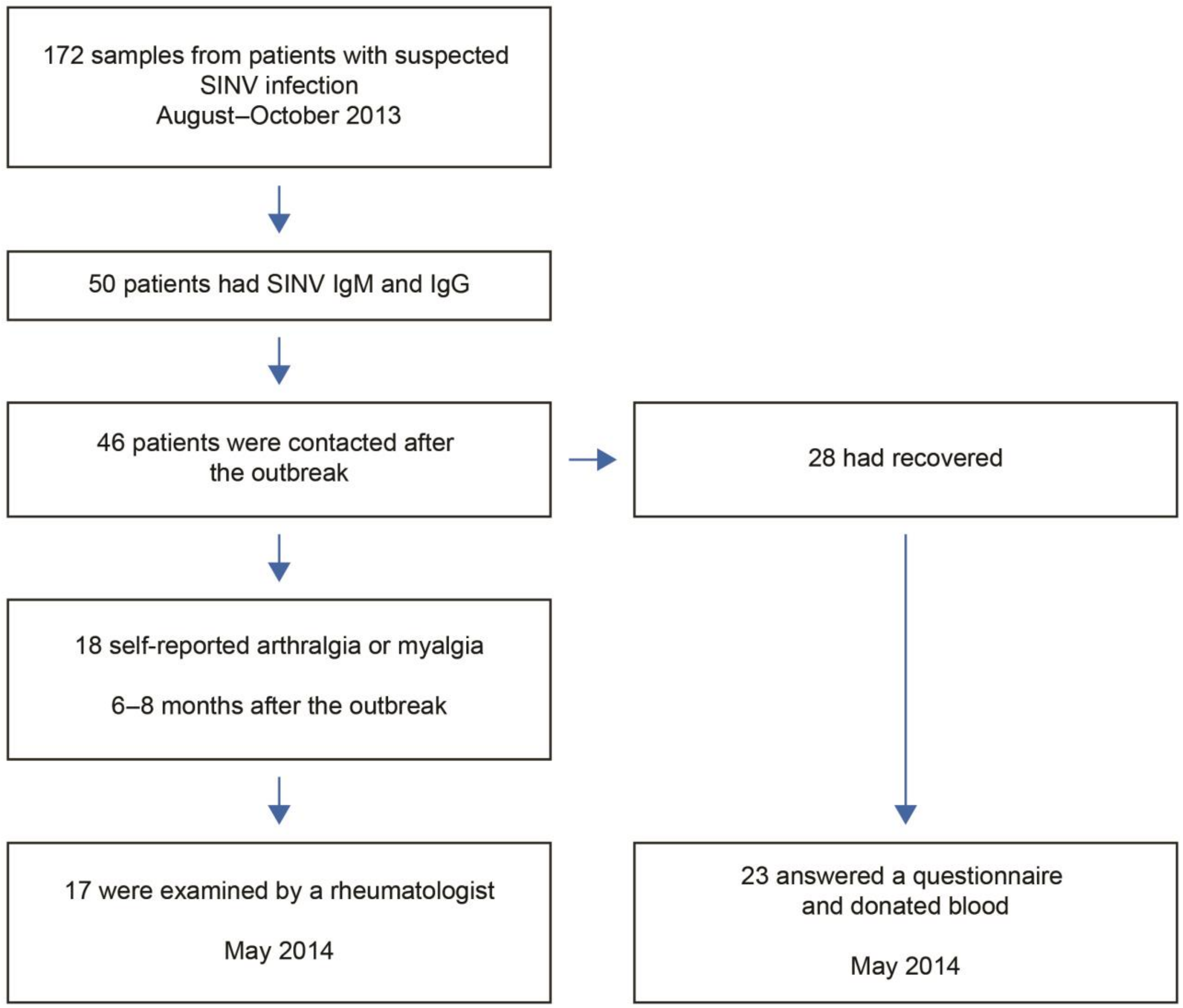

Technical Appendix Figure 1. Flowchart of patient interactions in study of Sindbis virus, Sweden, 2013.

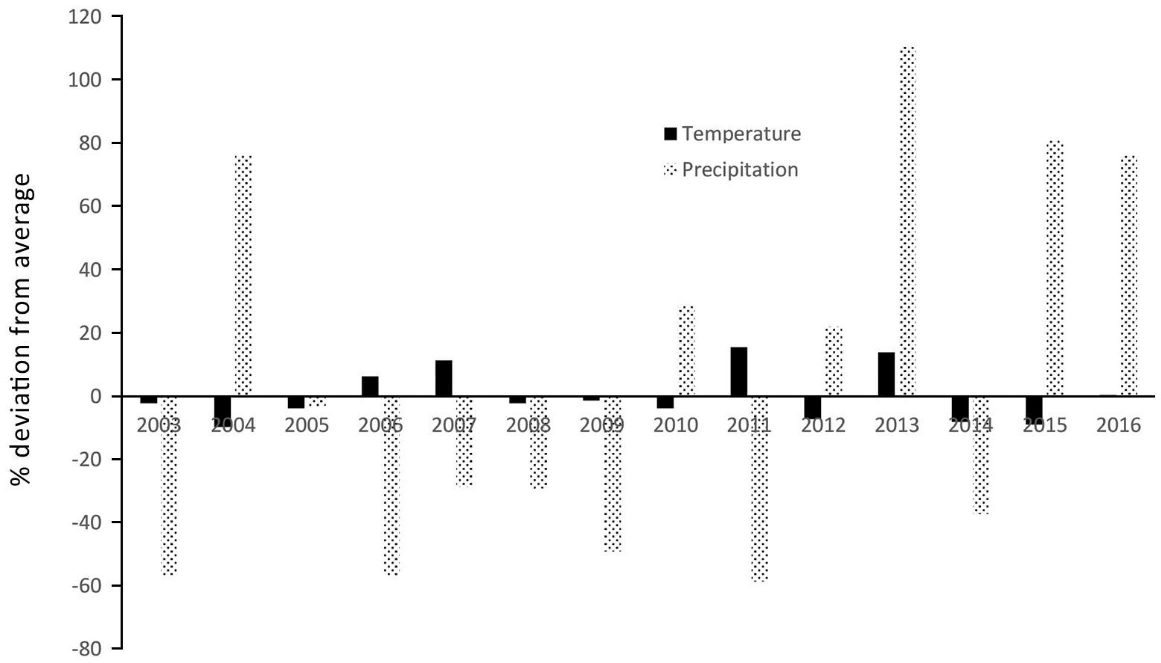

Technical Appendix Figure 2. High precipitation and high temperature in June at the weather station Bjuröklubb klubb (64°48′ N; 21°58′ E) in the middle of the outbreak area.
